# Metronidazole and Secnidazole Carbamates: Synthesis, Antiprotozoal Activity, and Molecular Dynamics Studies

**DOI:** 10.3390/molecules25040793

**Published:** 2020-02-12

**Authors:** Genaro Rocha-Garduño, Norma Angélica Hernández-Martínez, Blanca Colín-Lozano, Samuel Estrada-Soto, Emanuel Hernández-Núñez, Fernando Daniel Prieto-Martínez, José L. Medina-Franco, Juan Bautista Chale-Dzul, Rosa Moo-Puc, Gabriel Navarrete-Vázquez

**Affiliations:** 1 Facultad de Farmacia, Universidad Autónoma del Estado de Morelos, Cuernavaca, Morelos 62209, Mexico; azrg90@gmail.com (G.R.-G.); norma.hernandezm@uaem.edu.mx (N.A.H.-M.); clbi_ff@uaem.mx (B.C.-L.); enoch@uaem.mx (S.E.-S.); 2 Cátedra CONACyT, Departamento de Recursos del Mar, Centro de Investigación y de Estudios Avanzados del IPN, Unidad Mérida, Yucatán 97310, Mexico; emanuel.hernandez@cinvestav.mx; 3 Facultad de Química, Departamento de Farmacia, Universidad Nacional Autónoma de México, México City 04510, Mexico; ferdpm4@hotmail.com (F.D.P.-M.); medinajl@unam.mx (J.L.M.-F.); 4 Laboratorio de Apoyo a la Vigilancia Epidemiológica, Hospital de Especialidades 1, Centro Médico Nacional Ignacio García Téllez, Instituto Mexicano del Seguro Social, Mérida 97150, Yucatán, Mexico; jchaledzul@gmail.com; 5 Unidad de Investigación Médica Yucatán, Unidad Médica de Alta Especialidad, Centro Médico Nacional Ignacio García Téllez, Instituto Mexicano del Seguro Social, Mérida 97000, Yucatán, Mexico; moopuc@gmail.com

**Keywords:** carbamates, metronidazole, molecular dynamics, parasites, secnidazole

## Abstract

We prepared a series of 10 carbamates derivatives based on two common antiprotozoal drugs: metronidazole (**1**–**5**) and secnidazole (**6**–**10**). The compounds were tested in vitro against a set of two amitochondriate protozoa: *Giardia duodenalis* and *Trichomonas vaginalis*. Compounds **1**–**10** showed strong antiprotozoal activities, with potency values in the low micromolar-to-nanomolar range, being more active than their parent drugs. Metronidazole carbamate (**1**) was the most active of the series, with nanomolar activities against *G. duodenalis* (IC_50_ = 460 nM) and *T. vaginalis* (IC_50_ = 60 nM). The potency of compound **1** was 10 times greater than that of metronidazole against both parasites. None of compounds showed in vitro cytotoxicity against VERO cells tested at 100 µM. Molecular dynamics of compounds **1**–**10**, secnidazole, and metronidazole onto the ligand binding site of pyruvate–ferredoxin oxidoreductase of *T. vaginalis* and the modeled β-tubulin of *G. duodenalis* revealed putative molecular interactions with key residues in the binding site of both proteins implicated in the mode of action of the parent drugs.

## 1. Introduction

**Figure 1:**
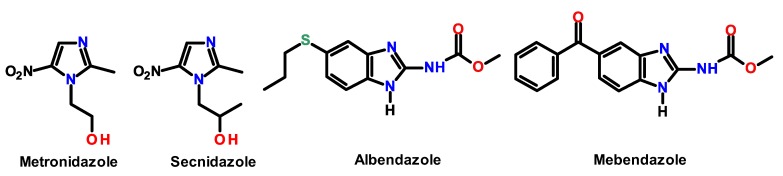
Most common antiprotozoal 5-nitroimidazole and benzimidazole methylcarbamate drugs of choice.

*Giardia duodenalis* is an intestinal parasite that infects several mammalian hosts including humans, and it is considered a leading cause of waterborne diarrheal disease and malabsorption syndrome [1]. *Trichomonas vaginalis* is the etiologic agent of the most common non-viral sexually transmitted disease in humans [2]. The chemotherapy against giardiasis and trichomoniasis is based on the use of 5-nitroimidazole drugs [3], such as metronidazole and secnidazole (Figure 1).

Metronidazole and secnidazole are prodrugs that are reductively activated (the nitro group is reduced) under low oxygen tension to produce free radical intermediates that form adducts with numerous biomolecules such as nucleic acids, proteins, and membrane lipids [4]. However, the clinical resistance to this chemotherapy reveals the need to search for novel and improved antiprotozoal drugs [4,5]. The mode of action of 5-nitroimidazoles is multifactorial [6], but one of the most accepted mechanisms is inhibition of the pyruvate–ferredoxin oxidoreductase (PFOR) [7]. Several benzimidazoles [8,9] and carbamates, such as albendazole and mebendazole, have shown giardicidal effects through the inhibition of β-tubulin polymerization [10]. In an effort to improve the antiprotozoal activity of this 5-nitroimidazole, we suggest the modification of its alcohol tail by several carbamates, increasing the lipophilicity of compounds and exploring the participation of the carbamate in the activity through the potential inhibition of β-tubulin polymerization plus the inhibition of PFOR. Thus, the in vitro antiparasitic effects of 10 newly designed carbamates on intestinal protozoa *G. duodenalis*, urogenital tract protozoa *T. vaginalis*, the cytotoxicity over mammalian VERO cells, and the molecular docking and dynamics prediction of this mode of binding over PFOR and β-tubulin are reported in this work.

## 2. Results and Discussion

### 2.1. Chemistry

**Scheme 1:**
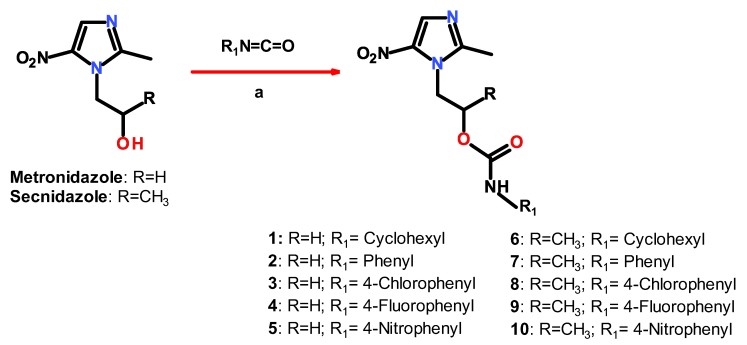
Synthesis of carbamates **1**–**10**: (**a**) triethylamine, NH_4_Cl (cat.), toluene, reflux.

Compounds **1**–**10** were synthesized starting from metronidazole (**11**) and secnidazole (**12**), which were reacted with appropriated isocyanates **13**–**17** (Scheme 1). Title compounds were recovered with 51–95% yields and were purified by recrystallization. In the ^1^H NMR spectra, we assigned the signals of the respective protons of the carbamate derivatives **1**–**10** on the basis of their chemical shifts, multiplicities, and coupling constants. All compounds showed a typical single signal ranging from 7.93 to 8.80 ppm, attributed to H4 of the imidazole ring. Another simple signal fluctuating from 2.05 to 2.48 ppm was assigned to the methyl group attached at position two of the imidazole. For the ^13^C NMR spectra, constant signals were found for the imidazole heterocycle: one signal at 150.1–156.8 ppm, attributed to C2, and two signals at 128.7–138.3 and 138.5–146.1 ppm, assigned to C4 and C5, respectively. Another recurrent signal was found in downfield shifts from 151.1 to 161.1 ppm, belonging to the carbamate carbonyl group. The methyl group attached at position 2 of imidazole always appeared at 14.3–19.1 ppm.

### 2.2. In Vitro Antiprotozoal Activity

The inhibition of in vitro growth of the amitochondriates *Giardia duodenalis* and *Trichomonas vaginalis* by compounds **1**–**10** is summarized in Table 1.

The biological activity of carbamates **1**–**10** was compared with the activity of the two parent antiprotozoal drugs of choice: metronidazole and secnidazole (Figure 1), which are commercial drugs used for standard therapies. All the carbamates showed strong giardicidal activity, with potency values oscillating from the low micromolar to the nanomolar range, being more active than or equipotent to their parent drugs. Compound **1** (cyclohexylcarbamate of metronidazole) and compounds **2** and **10** (phenylcarbamate of metronidazole and 4-nitrophenylcarbamate of secnidazole, respectively), were the most potent of the series (IC_50_ ranging from 0.46 to 0.96 µM) against *G. duodenalis.* They were almost 5–10 times more potent than metronidazole and secnidazole, which were equipotent among them. All carbamates were much more lipophilic than metronidazole and secnidazole, with a calculated MlogP around 1.68–3.87. This physicochemical property is important for high permeability of compounds across the protozoal membrane [11]. In our studies of *T. vaginalis,* compounds **1** and **2** also exhibited nanomolar trichomonicidal effects (IC_50_ = 60 and 90 nM, respectively). They were 10- to 15-fold more potent than metronidazole, which is the drug of choice for trichomoniasis. Secnidazole was the least potent compound against *T. vaginalis*, but all its carbamate derivatives **6**–**10** were more potent than this parent drug. This result is remarkable since if *Trichomonas* is more resistant to secnidazole treatment, its carbamate derivatives could be a possible therapeutic option for this protozoosis. In summary, the preliminary structure–activity relationship (SAR) analysis revealed that cyclic unsubstituted metronidazole carbamates **1** and **2** are the most potent compounds against both parasites. Of note, the SAR derived from the results in Table 1 is based on the biological activity measured with the entire parasite and not with isolated molecular targets (see Section 2.3).

### 2.3. In Vitro Cytotoxicity Assay

Compounds **1**–**10**, metronidazole, and secnidazole were evaluated for their intrinsic toxicity against mammalian VERO cell lines (Table 1), showing very low median cytotoxic concentration (CC_50_ > 100 µM). The selectivity index (SI) is the ratio of cytotoxicity to biological activities. If SI is greater than 10, it is typically an indicator that the underlying antiprotozoal activity is not due to the intrinsic cytotoxicity of a given compound [12]. Compounds **1**–**10** showed nanomolar to micromolar giardicidal activities and no observable cytotoxic effects at a 100 µM concentration, showing selectivity indexes prominently higher than 20. This implies that carbamates **1**–**10** are more selectively toxic against *G. duodenalis* than against mammalian cells. The same parasiticidal discrimination was observed between *T. vaginalis* and VERO cells.

### 2.4. Molecular Docking and Dynamics Studies

Based on the in vitro antiparasitic assays, the most active compounds (**1** and **2**) were selected for further computational studies to explore their putative mechanism of action at the molecular level. Molecular modeling studies were performed with two antiprotozoal relevant molecular targets: PFOR and β-tubulin. These two targets were chosen based on the known relationship with the parent compounds. A preliminary molecular docking simulation was performed to assess the putative binding mode of compounds **1** and **2** with the proposed targets. Then, molecular dynamics simulations were conducted to determine the relative stability of in silico binding modes. We emphasize that the molecular modeling studies reported in this work are intended to hypothesize the ligand–target interactions with two putative molecular targets. However, we did not intend to provide a detailed explanation for experimental SAR based on the predicted binding models. This is because it is not feasible to establish a reliable correlation between the measured antiprotozoal activity in vitro using the entire parasite, with the binding models derived with isolated 3D coordinates of the putative and isolated molecular targets. In vitro experiments have a significantly larger number of variables that cannot be addressed with in silico studies.

#### 2.4.1. Docking

**Figure 2:**
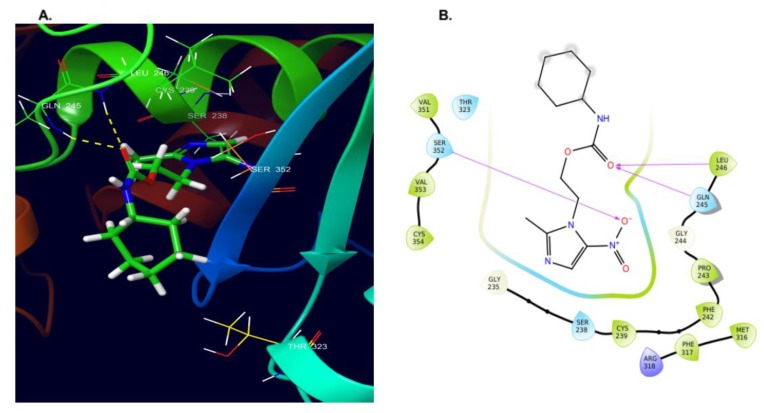
(**A**) 3D and (**B**) 2D representation of the binding model of compound **1** in the vicinity of the colchicine binding site of β-tubulin.

**Figure 3:**
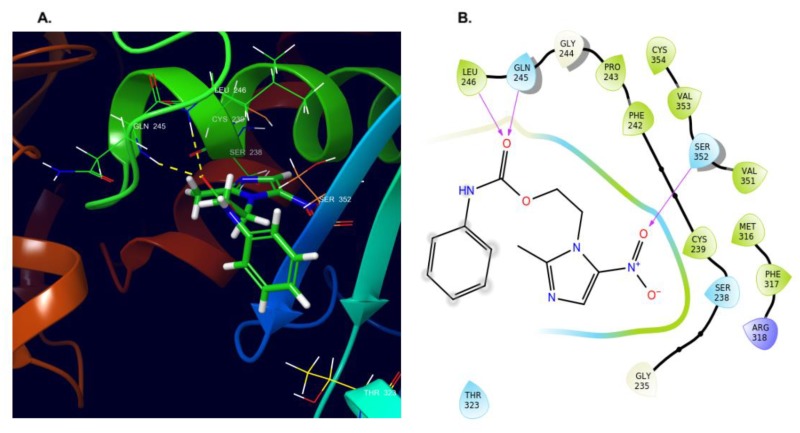
(**A**) 3D and (**B**) 2D representations of the binding model of compound **2** in the vicinity of colchicine binding site of β-tubulin.

Molecular docking suggested that compounds **1** and **2** have the potential to internalize in the vicinity of the colchicine binding site [13] of β-tubulin. In the binding models, both compounds form hydrogen bonds interactions with Gln-245, Ser-352, and form π-sp^3^ interactions with Leu-246 (Figure 2 and Figure 3). In addition, the predicted binding poses of **1** and **2** are characterized by several hydrophobic contacts with Cys-239, Phe-242, and Pro-243. Compound **1** showed a polar contact with Ser-238 (Figure 2), which may be due to the higher flexibility of the cyclohexane, facilitating a better fit in the binding cavity for the imidazole ring.

**Figure 4:**
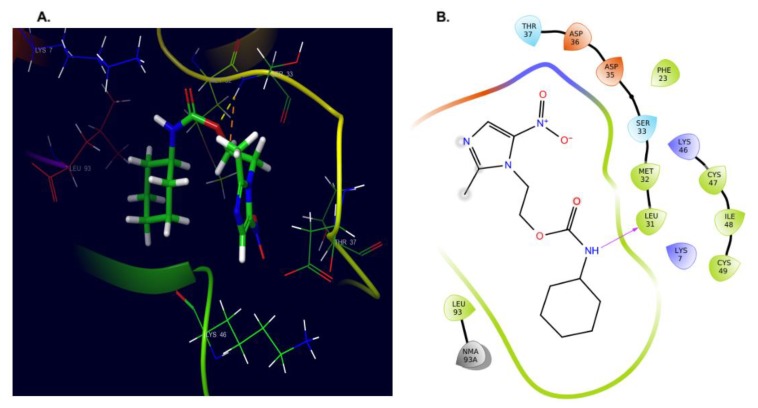
(**A)** 3D and (**B**) 2D representations of the binding model of compound **1** in the proposed site for metronidazole in pyruvate–ferredoxin oxidoreductase (PFOR).

**Figure 5:**
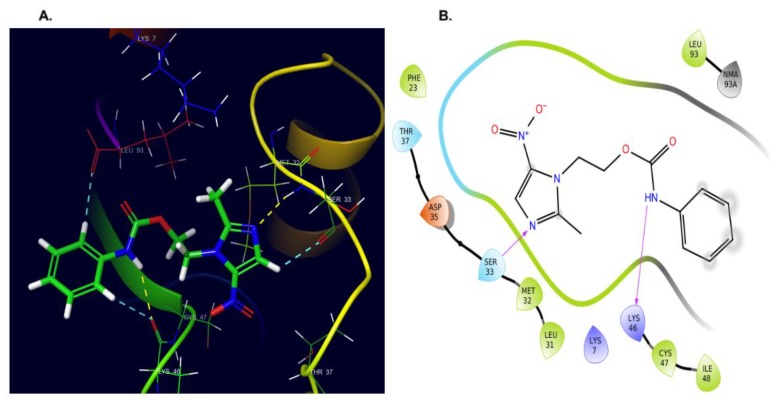
(**A**) 3D and (**B**) 2D representations of the binding model of compound **2** in the proposed site for metronidazole in PFOR.

For PFOR, we studied the previously proposed site for metronidazole [14], around Thr-37, close to the catalytic site. Docking simulations showed that metronidazole and the newly designed compounds have a suitable orientation for the binding site, this is, with the nitro group pointing toward the [2Fe–2S] core. Additionally, the scoring of compounds **1** and **2** was significantly better than that of metronidazole due to a better fit on the site (see Supplementary Materials). The main protein–ligand contacts of both compounds were with Leu-31, Met-32, Ser-33, Asp-36, Lys-7, and Lys-46 (Figure 4 and Figure 5). Docking poses provide a plausible explanation for the lower activity against *T. vaginalis* of bulky-substituted compounds on the R-position, as the size of the site could prevent the proper orientation of the 5-nitroimidazole scaffold.

#### 2.4.2. Dynamics

**Figure 6:**
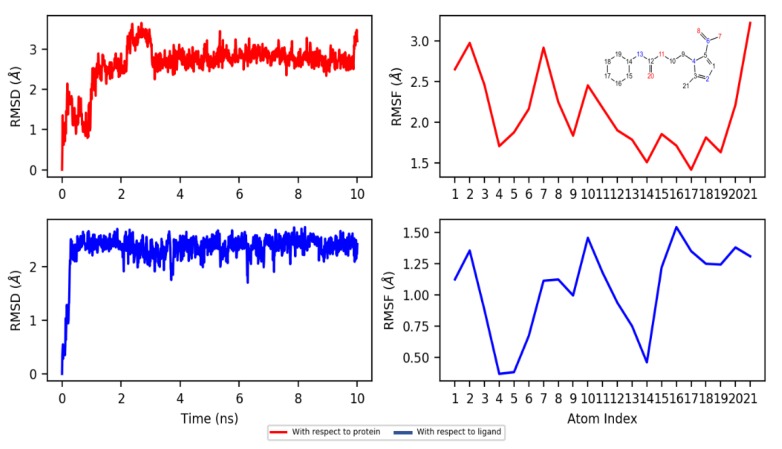
Dynamics of the top pose of compound **1** in the colchicine site of β-tubulin. Root mean square deviation (RMSD, left) and root mean square fluctuation (RMSF, right) are shown with respect to the protein fit (red) and the initial pose (blue).

**Figure 7:**
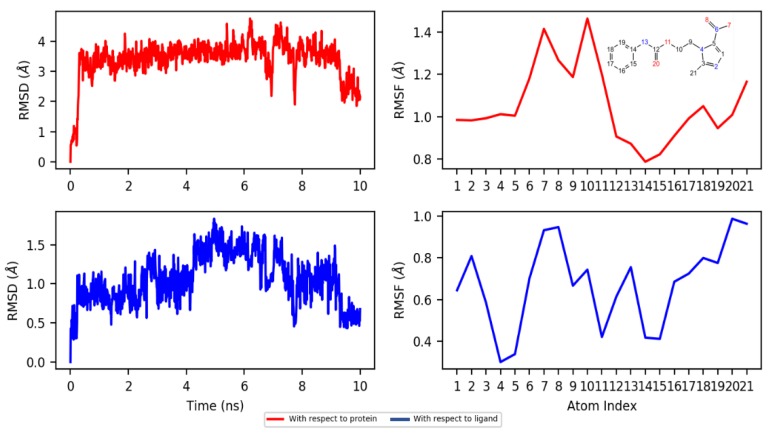
Dynamics of the top pose of compound **2** in the colchicine site of β-tubulin. RMSD (left) and RMSF (right) with respect to protein fit (red) and the initial pose (blue).

**Figure 8:**
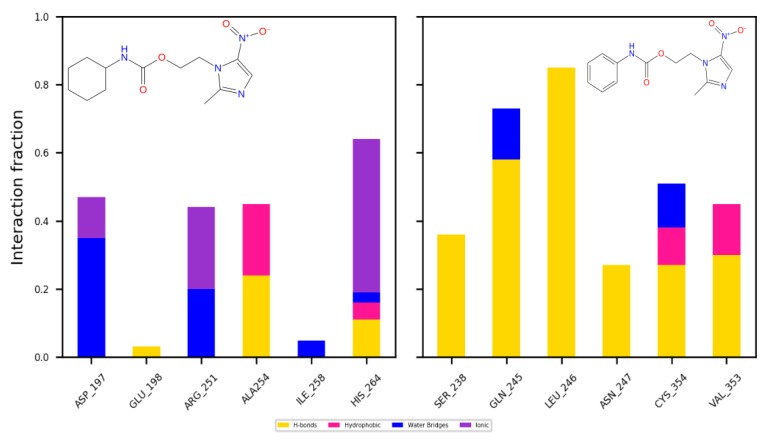
Protein–ligand interactions determined from the molecular dynamics of β-tubulin/nitroimidazole complexes: H-bond (yellow), hydrophobic (pink), water bridges (blue), and ionic (violet) interactions.

Langevin dynamics were simulated to assess the stability of the binding poses obtained with docking and the persistence of residue contacts over time. Langevin dynamics were chosen due to their physics, as its equations introduce dissipation and fluctuation terms, which account for multi-body contributions to the interaction [15]. Thus, Langevin dynamics are suited for accurate description of non-equilibrium and diffusion processes [16]. Langevin dynamics showed that protein–ligand complexes of compounds **1** and **2** in the colchicine site of β-tubulin are quite stable (Figure 6 and Figure 7). When residue contacts were examined, we found compound **1** shows different contacts when compared to docking pose (Figure 8). This is in agreement with the higher fluctuations of the 5-nitroimidazole ring, which changed its orientation towards Glu-197 and His-264. This observation further supports the proposed arrangement by Aguayo-Ortíz et al. [10]. During the dynamics simulations, compound **2** maintains H-bond contacts with Ser-238, Gln-245, Leu-246, and Asn-247. On such a short time scale, the compounds showed different orientations from a similar starting point. Considering the observed difference in IC_50_ values, a possible explanation is that both compounds need to move to the nocodazole site; in this case, the higher conformational freedom of the cyclohexane ring could provide a significant advantage. Further testing is require to confirm these observations, starting with longer simulation times to see if a similar behavior is shown for compound **2**. Future experimental testing to corroborate the in silico studies should include binding assays of compounds **1** and **2** with the proposed molecular targets, but these experiments were beyond the focus of this work on in vitro antiparasitic activity.

**Figure 9:**
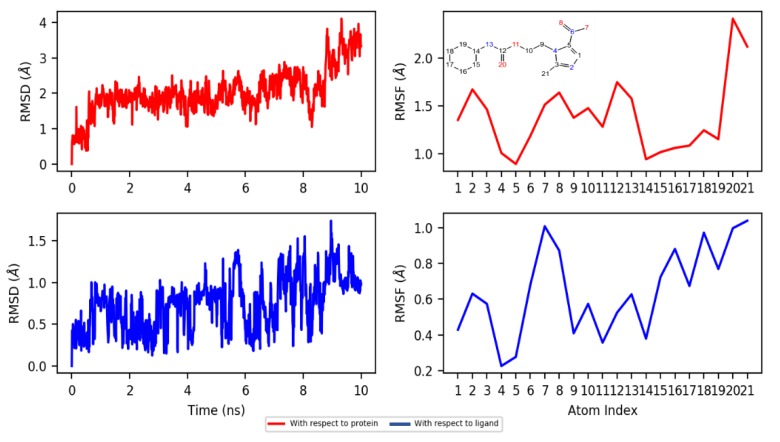
Dynamics of the top pose of compound **1** in the ligand recognition site of PFOR. RMSD (left) and RMSF (right) are shown with respect to protein fit (red) and the initial pose (blue).

**Figure 10:**
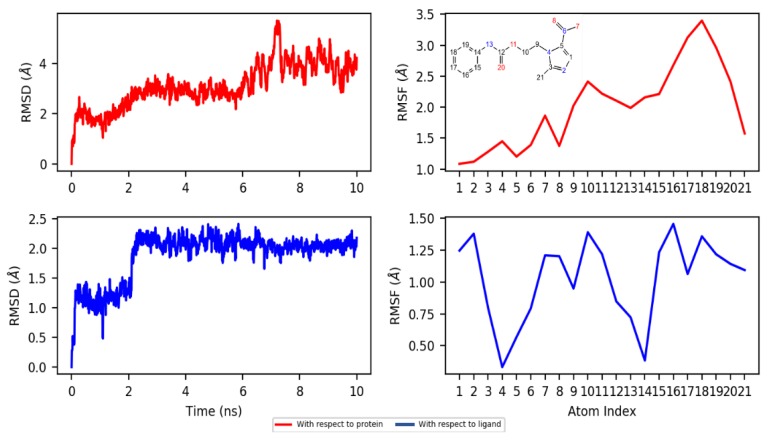
Dynamics of the top pose of compound **2** in the ligand recognition site of PFOR. RMSD (left) and RMSF (right) are shown with respect to protein fit (red) and the initial pose (blue).

For binding poses with PFOR, compound **1** showed less movement and fluctuations compared to **2**. In docking, **1** showed a better fit with PFOR compared to **2**. This result is mainly due to the planarity of the benzene ring, which is the group showing higher fluctuations over the course of the simulations (Figure 9 and Figure 10). The simulations showed that both compounds maintain contact with Ser-33 via a H-bond.

**Figure 11:**
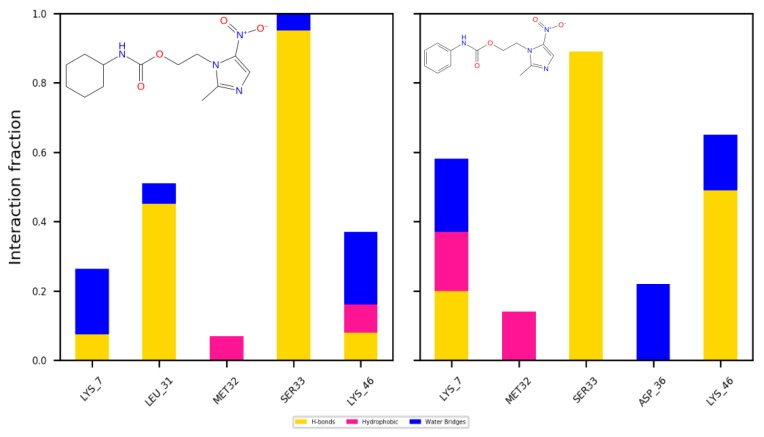
Protein–ligand interactions as obtained from molecular dynamics of PFOR/nitroimidazole complexes: H-bond (yellow), hydrophobic (pink), and water bridges (blue) interactions.

The main differences between **1** and **2** are the conserved contact with Lys-31 in the former and a higher contact rate with Lys-7 and -46 in the latter (Figure 11). Based on these observations, Ser-33 seems to be the main contact for proper orientation with the site; however, this interaction was only observed for **1** in a dynamic setting.

## 3. Materials and Methods 

### 3.1. Chemistry

Solvents and reagents were acquired from Sigma-Aldrich (St. Louis, MO, USA). Melting points were obtained using a capillary apparatus from Stanford Research Systems (Sunnyvale, CA, USA). All reactions were monitored by thin layer chromatography (TLC) on 0.2 mm precoated silica gel 60 F_254_ Merck plates (Kenilworth, NJ, USA). ^1^H NMR spectra were determined on Varian 600 MHz AR Premium Compact (Varian-Agilent, Santa Clara, CA, USA) and ^13^C NMR (150 MHz) instrument (Varian-Agilent, Santa Clara, CA, USA). Chemical shifts are reported in ppm in DMSO-d_6_ and CDCl_3_ as deuterated solvents. Mass spectrometry was obtained from a JEOL JMS-700 spectrometer by electronic impact (JEOL, Tokyo, Japan).

### 3.2. General Procedure for the Synthesis of Compounds **1**–**10**

To a solution of metronidazole or secnidazole (0.0023 mol) in toluene (5 mL), we dropwise added the suitable isocyanate **13**–**17** (0.0046 mol, 2 equivalents), 10% triethylamine, and NH_4_Cl as catalysts at 25 °C. The mixture was stirred at reflux (110 °C) under a nitrogen atmosphere for 7–33 h. The solvent was removed using a high vacuum system, and the residue was suspended in cold water. The solids were recovered by filtration, dried in the hood and recrystallized from suitable solvent.

**2-(2-methyl-5-nitro-1*H*-imidazol-1-yl)ethyl cyclohexylcarbamate(1)**: 21 h, yield 67%, recrystallized from ethanol-water, white crystals, Mp 103.7 °C (dec.). ^1^H NMR (600 MHz, CDCl_3_) δ: 1.10–1.31 (m, 4H, H-2’, H-6´), 1.57–1.68 (m, 2H, H-4´), 1.68–1.87 (m, 4H, H-3´, H-5´), 2.45 (s, 3H, CH_3_), 3.39–3.41 (m, 1H, H-1´), 4.35 (t, 2H, N–CH_2_), 4.54 (t, 2H, O–CH_2_), 7.93 (s, 1H, H-4), 9.32 (bs, 1H, *N*–H) ppm. ^13^C NMR (150 MHz, CDCl_3_) δ: 14.19 (CH_3_), 24.7 (C-3´, C-5´), 25.4 (C-4´), 33.2 (C-2´, C-6´), 45.6 (N–CH_2_), 50.04 (C-1´), 62.4 (O–CH_2_), 133.1 (C-4), 138.5 (C-5), 150.8 (C-2), 154.6 (C=O) ppm. MS/EI: *m/z* (% int. rel). 296.32 (M^+,^ 10%), 225.20 (M-71, 100%).

**2-(2-methyl-5-nitro-1*H*-imidazol-1-yl)ethyl phenylcarbamate(2)**: 15 h, yield 86%, recrystallized from ethanol-water, white powder Mp 197.7 °C (dec.). ^1^H NMR (600 MHz, DMSO-d6) δ: 2.48 (s, 3H, CH_3_), 4.47 (t, 2H, N–CH_2_), 4.63 (t, 2H, O–CH_2_), 7.01 (t, 1H, H-4´), 7.27 (t, 2H, H-3´, H-5´, *Jo =* 7.4 Hz), 7.40 (d, 2H, H-2´, H-6´, *Jo =* 7.6 Hz), 8.06 (s, 1H, H-4), 9.66 (bs, 1H, *N*–H) ppm. ^13^C NMR (150 MHz, DMSO-d6) δ: 19.1 (CH_3_), 50.6 (N–CH_2_), 67.4 (O–CH_2_), 123.7 (C-4´), 127.8 (C-2´, C-6´), 133.9 (C-3´, C-5´), 138.3 (C-4), 143.7 (C-5), 143.9 (C-1´), 156.8 (C-2), 158.12 (C=O) ppm. MS/EI: *m/z* (% int. rel). 290.27 (M^+,^ 80%), 170.05 (M-120, 100%).

**2-(2-methyl-5-nitro-1*H*-imidazol-1-yl)ethyl (4-chlorophenyl)carbamate(3):** 7 h, yield 65%, recrystallized from ethanol, yellow crystals, Mp 175.0–177.3 °C; ^1^H NMR (600 MHz, DMSO-d6) δ: 2.45 (s, 3H, CH_3_), 4.45 (t, 2H, N–CH_2_), 4.58 (t, 2H, O–CH_2_), 7.29 (d, 2H, H-2´, H-6´, *Jo =* 8.9 Hz), 7.39 (d, 2H, H-3´, H-5´, *Jo =* 8.9 Hz), 8.02 (s, 1H, H-4), 9.77 (bs, 1H, *N*–H) ppm. ^13^C NMR (150 MHz, DMSO-d6) δ: 14.4 (CH_3_), 45.7 (N–CH_2_), 62.82 (O–CH_2_), 120.4 (C-2´, C-6´), 129.1 (C-3´, C-5´), 133.5 (C-1´), 138.2 (C-4), 138.9 (C-5), 152.1 (C-2), 153.3 (C=O) ppm. MS/EI: *m/z* (% int. rel). 324.71 (M^+,^ 100%), 325.16 (M+2, 33%).

**2-(2-methyl-5-nitro-1*H*-imidazol-1-yl)ethyl (4-fluorophenyl)carbamate** (**4**): 9 h, yield 77%, recrystallized from ethanol-water, yellow crystals, Mp 168.2–170.6 °C; ^1^H NMR (600 MHz, DMSO-d6) δ: 3.34 (s, 3H, CH_3_), 4.46 (t, 2H, N–CH_2_), 4.60 (t, 2H, O–CH_2_), 7.13 (d, 2H, H-2´, H-6´, *Jo =* 8.6 Hz), 7.10 (d, 2H, H-3´, H-5´, *Jo =* 8.6Hz), 8.02 (s, 1H, H-4), 9.63 (bs, 1H, *N*–H) ppm. ^13^C NMR (150 MHz, DMSO-d6) δ: 14.4 (CH_3_), 45.8 (N–CH_2_), 62.7 (O–CH_2_), 115.7 (d, C-3´, C-5´, ^2^J_C-F_ = 21 Hz), 120.6 (C-1), 133.5 (C-2´, C-6´), 135.4 (C-5), 138.9 (C-4), 152.07 (C-2), 153.5 (C=O), 158.2 (d, C-4´, ^1^J_C-F_ = 237.6 Hz). MS/EI: *m/z* (% int. rel). 308.26 (M^+,^ 10%), 137.13 (M-171, 100%).

**2-(2-methyl-5-nitro-1*H*-imidazol-1-yl)ethyl (4-nitrophenyl)carbamate(5):** 10 h, yield 87%, recrystallized from ethanol, yellow crystals, Mp 253.9–256.0 °C. ^1^H NMR (600 MHz, DMSO-d6) δ: 2.48 (s, 3H, CH_3_), 4.50 (t, 2H, N–CH_2_), 4.85 (t, 2H, O–CH_2_), 7.62 (d, 2H, H-2´, H-6´, *Jo =* 9.8 Hz), 8.14 (s, 1H, H-4), 8.28 (d, 2H, H-3´, H-5´, *Jo =* 9.8 Hz), 9.69 (bs, 1H, *N*–H) ppm. ^13^C NMR (150 MHz, DMSO-d6) δ: 14.2 (CH_3_), 44.5 (N–CH_2_), 61.9 (O–CH_2_), 117.6 (C-2´, C-6´), 124.6 (C-3´, C-5´), 129.4 (C-4), 138.5 (C-5), 139.4 (C-1´), 141.2 (C-4´), 150.1 (C-2), 151.1 (C=O) ppm. MS/EI: *m/z* (% int. rel). 335.27 (M^+,^ 100%), 125.96 (M-209, 20%).

**1-methyl-2-(2-methyl-5-nitro-1*H*-imidazol-1-yl)ethyl cyclohexylcarbamate** (**6**): 21 h, yield 51%, recrystallized from ethanol-water, white crystals, Mp 219.2–222.7 °C. ^1^H NMR (600 MHz, DMSO-d6) δ: 1.10–1.26 (m, 4H, H-2´, H-6´), 1.22 (d, 3H, CH_3_), 1.46–1.49 (m, 2H, H-4´), 1.57–1.71 (m, 4H, H-3´, H-5´), 2.42 (s, 3H, CH_3_), 4.18-4.22 (m, 1H, H-1´), 4.48 (d, 2H, N-CH_2_), 5.04–5.07 (m, 1H, O-CH), 7.93 (s, 1H, H-4), 9.32 (bs, 1H, *N*–H) ppm. ^13^C NMR (150 MHz, DMSO-d6) δ: 14.4 (CH_3_), 17.9 (CH_3_), 25.4 (C-4´), 25.8 (C-3´, C-5´), 33.1 (C-2´, C-6´), 48.6 (N-CH_2_), 50.1 (C-1´), 64.7 (O-CH), 128.8 (C-4), 134.6 (C-5), 153.4 (C-2), 155.7 (C=O) ppm. MS/EI: *m/z* (% int. rel). 310.34 (M^+,^ 1%), 264.13 (M-46, 20%), 139.11 (M-171, 100%).

**1-methyl-2-(2-methyl-5-nitro-1*H*-imidazol-1-yl)ethyl phenylcarbamate (7):** 33 h, yield 19%, recrystallized from ethanol-water, white crystals, Mp 238.9–240.5 °C. ^1^H NMR (600 MHz, DMSO-d6) δ: 1.31 (d, 3H, CH_3_), 2.47 (s, 3H, CH_3_), 4.57 (d, 2H, N–CH_2_), 5.18-5.20 (m, 1H, O–CH), 6.92-695 (m, 1H, H-4´), 7.23–7.26 (m, 2H, H-3´-H5´), 7.42 (dd, 2H, H-2´, H,6´, *J*m = 1.14, *J*o= 8.64 Hz), 7.99 (s, 1H, H-4), 9.52 (bs, 1H, *N*–H) ppm. ^13^C NMR (150 MHz, DMSO-d6) δ: 14.5 (CH_3_), 17.9 (CH_3_), 46.9 (N–CH_2_), 59.8 (O–CH), 118.3 (C-2´, C-6´), 122.3 (C-4´), 129.2 (C-3´-C-5´), 133.5 (C-4), 138.6 (C-1´), 140.1 (C-5), 152.9 (C-2), 161.1 (C=O) ppm. MS/EI: *m/z* (% int. rel). 304.30 (M^+,^ 20%), 258.08 (M-46, 30%), 139.11 (M-165, 100%).

**1-methyl-2-(2-methyl-5-nitro-1*H*-imidazol-1-yl)ethyl (4-chlorophenyl)carbamate (8):** 17 h, yield 91%, recrystallized from acetone, white crystals, Mp 268.1 °C (dec.). ^1^H NMR (600 MHz, DMSO-d6) δ: 1.03 (d, 3H, CH_3_), 2.05 (s, 3H, CH_3_), 4.33 (d, 2H, N–CH_2_), 5.85-5.87 (m, 1H, O–CH), 7.29(dd, 2H, H-2´–H-6´, *J*m = 2.22, *J*o = 8.88 Hz), 7.45 (dd, 2H, H-3´–H-5´, *J*m = 2.22, *J*o = 8.94 Hz), 8.80 (s, 1H, H-4), 9.52 (bs, 1H, *N*-H) ppm. ^13^C NMR (150 MHz, DMSO-d6) δ: 14.5 (CH_3_), 17.9 (CH_3_), 50.4 (N–CH_2_), 69.4 (O–CH), 120.5 (C-2´, C-6´), 126.8 (C-4´), 129.1 (C-3´-C-5´), 133.5 (C-4), 138.2 (C-1´), 139.1 (C-5), 152.1 (C-2), 152.9 (C=O) ppm. MS/EI: *m/z* (% int. rel). 338.74 (M^+,^ 1%), 152.01 (M-186, 100%).

**1-methyl-2-(2-methyl-5-nitro-1*H*-imidazol-1-yl)ethyl (4-fluorophenyl)carbamate (9):** 20 h, yield 65%, recrystallized from ethanol-water, yellow crystals, Mp 162.3–163.5 °C. ^1^H NMR (600 MHz, DMSO-d6) δ: 1.31 (d, 3H, CH_3_), 2.06 (s, 3H, CH_3_), 4.56 (d, 2H, N-CH_2_), 5.16–5.21 (m, 1H, O–CH), 7.04–7.10 (m,2H, H-3´–H-5´), 7.42 (dd, 2H, H-2´–H-6´, *J*m = 2.4, *J*o = 9.18 Hz), 7.98 (s, 1H, H-4), 9.56 (bs, 1H, *N*–H) ppm. ^13^C NMR (150 MHz, DMSO-d6) δ: 14.5 (CH_3_), 17.9 (CH_3_), 50.4 (N–CH_2_), 69.2 (O–CH), 115.7 (d, C-3´, C-5´, ^2^*J*_C-F_ = 22.5 Hz), 120.5 (d, C-2´, C-6´, ^3^*J*_C-F_ = 9 Hz), 133.6 (C-4), 136.4 (d, C-1´, ^4^*J*_C-F_ = 3 Hz), 139.0 (C-5), 152.1 (C-2), 153.1 (C=O), 157.8 (d, C-4´, ^4^*J*_C-F_=237 Hz) ppm. MS/EI: *m/z* (% int. rel). 322.29 (M^+,^ 20%), 276.07 (M-46, 10%).

**1-methyl-2-(2-methyl-5-nitro-1*H*-imidazol-1-yl)ethyl (4-nitrophenyl)carbamate (10):** 15 h, yield 65%, recrystallized from ethanol, yellow crystals, Mp 235.4 °C (dec.). ^1^H NMR (600 MHz, DMSO-d6) δ: 1.34 (d, 3H, CH_3_), 2.05 (s, 3H, CH_3_), 4.58 (d, 2H, N–CH_2_), 5.23–5.26 (m, 1H, O–CH), 7.53 (dd, 2H, H-2´–H-6´, *J*m = 3.06, *J*o = 9.3 Hz), 7.98 (s, 1H, H-4), 8.18 (dd, 2H, H-3´–H-5´, *J*m = 3.1, *J*o = 9.3 Hz), 9.62 (bs, 1H, *N*–H) ppm. ^13^C NMR (150 MHz, DMSO-d6) δ: 14.5 (CH_3_), 17.8 (CH_3_), 50.22 (N–CH_2_), 7.01 (O–CH), 118.3 (C-2´, C-6´), 125.6 (C-3´–C-5´), 133.6 (C-4), 139.1 (C-1´), 142.3 (C-4´), 146.1 (C-5), 152.1 (C-2), 152.7 (C=O) ppm. MS/EI: *m/z* (% int. rel). 349.29 (M^+,^ 1%), 137.99 (M-211, 100%).

### 3.3. Biological Assays

#### 3.3.1. Giardicidal and Trichomonicidal Assays

*G. intestinalis* strain IMSS:0696:1 and *T. vaginalis* strain GT3 were cultured in TYI-S-33 medium, complemented with 10% calf serum and bovine bile [12]. In vitro susceptibility assays were executed using 4 × 10^4^ trophozoites of *G. intestinalis* or *T. vaginalis*, which were incubated at 37 °C for 48 h with cumulative concentrations of carbamates **1**–**10**, metronidazole, and secnidazole, and also incubated alone in culture medium, with DMSO used as the solvent (0.05%). Subsequently, trophozoites were washed and subcultured for another 48 h in fresh medium without any drugs. Once this time was reached, trophozoites were counted and the median inhibitory concentration (IC_50_) was calculated. All the experiments were completed in triplicate.

#### 3.3.2. Cytotoxicity on VERO Cell Line

We cultivated 1.5 × 10^4^ VERO cells in a 96-well plate and incubated in DMEM media complemented with 10% fetal bovine serum, 100 µg/mL streptomycin, and 100 UI/mL penicillin. The culture was incubated at 37 °C in a 5% CO_2_ atmosphere with 95% humidity for 48 h. When cells reached >80% confluence, the culture media were replaced and the VERO cells were treated with carbamates **1**–**10**, metronidazole, and secnidazole at 1–100 µM dissolved in DMSO at a maximum concentration of 0.05%. After 48 h of incubation, viability of the cells was estimated using the sulforhodamine B method [12]. The concentration of the compounds that killed 50% of the cells (CC_50_) was calculated by nonlinear fit (GraphPad Prism 4 software). All concentrations were evaluated in triplicate.

### 3.4. In Silico Methods

#### 3.4.1. Homology Modeling

The modeling was completed with YASARA (v. 19.12.14) [17], following a protocol detailed elsewhere [18]. Using sequence alignment and analysis [19,20], we identified the β-tubulin of *Bos taurus, Sus scrufa,* and *Ovis aries* as proper templates for the *G. duodenallis* tubulin model. The model was further refined with a short molecular dynamics simulation (500 ps) with the YASARA2 forcefield, which includes knowledge-based potentials. The simulation was conducted at 298 K and 1 atm (NPT ensemble), using an 8 Å cutoff for Van der Waals interactions. Coulombic interactions were computed using the particle mesh Ewald (PME) method [21]. The integration timestep was set at 2 fs, with a recording interval of 25 ps. Finally, the quality of the model was assessed by means of *Z*-score, QMEAN [22], and with the SAVES server (https://servicesn.mbi.ucla.edu/SAVES/), which includes PROCHECK [23], WHATCHECK [24], ERRAT [25], VERIFY 3D [26], and PROVE [27] metrics (see Supplementary Materials).

#### 3.4.2. Molecular Docking

The crystal structure of the PFOR (PDB-ID: 1L5P) at 2.2 Å resolution was obtained from the Protein Data Bank (http://www.rcsb.org/pdb) [28]. All docking was calculated with the Molecular Operating Environment (MOE, Chemical Computing Group Inc. Quebec, Canada, http://www.chemcomp.com) version 2019.01 [29]. All water molecules were deleted, and the hydrogen atoms and charges were adjusted with the PFROSST force field from the MOE suite. This forcefield uses AMBER parameters for protein description and MMFF94 for small organic molecules. The 3D structures were built and minimized in MOE; using the same force field as that mentioned above, partial charges were added with MOPAC using the AM1-BCC method [30]. As a validation procedure for the binding sites, metronidazole was blindly docked on both targets (see Supplementary Materials). As a placement function, Alpha Triangle was selected, and the scores were calculated with the GBVI/WSA scoring function, which measures the free energy of binding using forcefield parameters [31] and considers implicit solvation contribution [32]. After the confirmation of binding, the sites were defined around Cys-239 (β-tubulin) and Thr-37 (PFOR). The docking was performed considering all residues within a 5.0 Å sphere centered on the defined sites of each target. For each ligand, 10,000 conformations were generated prior to placement. The top 100 placements were refined by scoring function. After molecular docking, the best binding poses were visually inspected. Finally, graphical representations of ligand interactions were created in Maestro (Schrödinger, NY, USA). The top-ranked poses were selected for further analysis with molecular dynamics.

#### 3.4.3. Molecular Dynamics

Non-equilibrium Langevin dynamics were used to determine the putative stability of binding modes obtained from docking with Desmond [33].

Using the top poses of the most active compounds as starting point, protein–ligand complexes were prepared in Maestro (19-2), with the System Builder utility. The complexes were buffered in a truncated octahedron box with a 14.0 Å solvent shell around the protein, the system was neutralized, and NaCl was added to obtain a 0.15 M concentration. The system was parameterized with the OPLS 2005 force field. Each system was minimized in three steps, using Brownian dynamics under NVT ensemble at 10 K with a small time-step to avoid numerical errors [34], first with a 1000 kcal/mol/Å restraint on solute heavy atoms for 250 ps, followed by another 250 ps of Brownian dynamics in similar conditions with a 10 kcal/mol/Å restraint on the protein backbone. Finally, the simulation was conducted for 500 ps without restraints. Then the complexes were further relaxed by heating slowly from 10 to 300 K in the NVT ensemble for 500 ps using the Berendsen thermostat and a 2 fs timestep. This was followed by a 1000 ps relaxation in NPT (1 atm) ensemble using a Langevin thermostat and barostat (with a relaxation times of 0.02 and 1.0 ps, respectively). Production runs lasted 10 ns, which were repeated 10 times using different random seeds. Trajectories were analyzed with the simulation interaction diagram utility. Ligand RMSD, RMSF, and residue contacts from each run were compared to obtain the average behavior and the relative stability of the pose [35].

## 4. Conclusions

We reported the one-step preparation of 10 carbamate derivatives of metronidazole and secnidazole in modest yields, which showed strong nanomolar and micromolar antiprotozoal activity against two amitochondriate parasites, *Giardia duodenalis* and *Trichomonas vaginalis,* with no observable cytotoxic effects in mammalian VERO cells. The giardicidal effect of carbamates **1**–**10** was improved compared to the two first-line commercial drugs: metronidazole and secnidazole. All compounds showed trichomonicidal effects greater than secnidazole. The antiprotozoal effect could be related to the higher lipophilicity of the 10 compounds, because they could penetrate the protozoal membrane more effectively. The most active compounds were **1** and **2**, which are metronidazole cyclohexylcarbamate and phenylcarbamate, respectively. The plausible modes of action of compounds **1** and **2** involve inhibition of PFOR and β-tubulin as suggested by docking and molecular dynamics.

## Figures and Tables

**Table 1:** In vitro antiprotozoal and cytotoxic effects of carbamates **1**–**10** on *Giardia duodenalis* and *Trichomonas vaginalis.*

Compound	IC_50_ (µM) ^a^		CC_50_ (µM) ^a^	SI *=* CC_50_/IC_50_		MLogP
	*G. duodenalis*	*T. vaginalis*	VERO Cells	*G. duodenalis*	*T. vaginalis*	
**1**	0.46 ± 0.01	0.06 ± 0.01	>100	>217	>1666	3.73
**2**	0.98 ± 0.01	0.09 ± 0.01	>100	>102	>1111	1.72
**3**	1.14 ± 0.12	1.79 ± 0.12	>100	>87	>55	2.4
**4**	2.03 ± 0.01	3.22 ± 0.21	>100	>49	>31	1.89
**5**	2.43 ± 0.02	6.25 ± 0.12	>100	>41	>16	1.68
**6**	2.64 ± 0.61	8.64 ± 0.73	>100	>37	>11	3.87
**7**	1.67 ± 0.21	6.52 ± 0.71	>100	>59	>15	1.86
**8**	4.05 ± 0.24	5.93 ± 0.51	>100	>24	>16	2.54
**9**	3.23 ± 0.11	4.82 ± 1.08	>100	>30	>20	2.02
**10**	0.86 ± 0.09	9.99 ± 0.56	>100	>116	>10	1.82
**Metronidazole**	4.42 ± 0.23	0.93 ± 0.12	>100	>22	>107	−0.47
**Secnidazole**	4.11 ± 0.12	13.45 ± 1.23	>100	>24	>7	−0.10

^a^ Means of three experiments; IC_50_, median inhibitory concentration; CC_50_, median cytotoxic concentration; SI, selectivity index; MLogP, logarithm of the partition coefficient, calculated in https://www.molinspiration.com/cgi-bin/properties.

## Supplementary Materials

The following are available online at https://www.mdpi.com/1420-3049/25/4/793/s1, Figure S1. Multiple sequence alignment of β-tubulin from different organisms used in this study, Figure S2. Global quality measures of the homology model constructed; Figure S3. Additional quality assessment of the homology model. (A) Main Ramachandran plot. (B) 3D structure of homology model colored by quality. (C) QMEAN4 normalized score. (D) Local quality per-residue; Figure S4. Model quality obtained from SAVES server. (A) Residue distribution. (B) Verify 3D. (C) ERRAT; Figure S5. Docking pose of metronidazole in PFOR. (A) 3D and surface representation. (B) 2D residue contacts; Figure S6. Docking pose of metronidazole in β-tubulin. (A) 3D and surface representation. (B) 2D residue contacts; Table S1. Template scoring as obtained by PSI-BLAST and YASARA; Table S2. Docking scores of the top five poses of metronidazole and the most active compounds in the proposed binding site of PFOR; Table S3. Docking scores of the top five poses of metronidazole and the most active compounds in the proposed binding site of β-tubulin.
