# Supplementary material for: Metronidazole and Secnidazole Carbamates: Synthesis, Antiprotozoal Activity, and Molecular Dynamics Studies
Source: Molecules. 2020 Feb 12;25(4):793. doi: 10.3390/molecules25040793 (PMC7071106; doi:10.3390/molecules25040793)
Supplement: Supplementary file 1 [file molecules-25-00793-s001.pdf]

Metronidazole and Secnidazole Carbamates: Synthesis, antiprotozoal activity and molecular dynamics studies

Genaro Rocha-Garduño[1], Norma Angélica Hernández-Martinez[1], Blanca Colín-Lozano[1], Samuel Estrada-Soto[1], Emanuel Hernández-Núñez[2], Fernando Daniel Prieto-Martinez[3], José L. Medina-Franco[3], Juan Bautista Chale-Dzul[4], Rosa Moo-Puc[5], and Gabriel Navarrete-Vázquez[1,*]

[1] Facultad de Farmacia, Universidad Autónoma del Estado de Morelos, Cuernavaca, Morelos, 62209, México; azrg90@gmail.com (A. G. R.-G.) norma.hernandezm@uaem.edu.mx (N.A. H.-M.); clbi_ff@uaem.mx (B.C.-L); enoch@uaem.mx (S. E-S); gabriel_navarrete@uaem.mx (G.N.-V)

[2] Cátedra CONACyT, Departamento de Recursos del Mar, Centro de Investigación y de Estudios Avanzados del IPN, Unidad Mérida, 97310, Yucatán, México; emanuel.hernandez@cinvestav.mx (E.H.-N.)

[3] Facultad de Química, Departamento de Farmacia, Universidad Nacional Autónoma de México, México City 04510, México; ferdpm4@hotmail.com (F.D. P.-M.), medinajl@unam.mx (J. L. M.-F.)

[4] Laboratorio de Apoyo a la Vigilancia Epidemiológica, Hospital de Especialidades 1, Centro Médico Nacional Ignacio García Téllez, Instituto Mexicano del Seguro Social, 97150 Mérida, Yucatán, México. jchaledzul@gmail.com (J.B.C-D)

[5] Unidad de Investigación Médica Yucatán, Unidad Médica de Alta Especialidad, Centro Médico Nacional Ignacio García Téllez, Instituto Mexicano del Seguro Social, 97000, Mérida, Yucatán, México; moopuc@gmail.com (R. M.-P.)

*Correspondence e-mail: gabriel_navarrete@uaem.mx

Table of contents

**Contents**	**Page**
**Figure S1.** Multiple sequence alignment of β-tubulin from different organisms used in this study.	**1**
**Table S1.** Template scoring as obtained by PSI-BLAST and YASARA.	**2**
**Figure S2.** Global quality measures of the homology model constructed for *G. duodenalis* β-tubulin.	**2**
**Figure S3.** Additional quality assessment of the homology model. A) Main Ramachandran plot. B) 3D structure of homology model colored by quality. C) QMEAN4 normalized score. D) Local quality per-residue.	**3**
**Figure S4.** Model quality obtained from SAVES server. A) Residue distribution. B) Verify 3D. C) ERRAT.	**3**
**Figure S5.** Docking pose of metronidazole in PFOR. A) 3D and B) 2D representation with residue contacts.	**4**
**Figure S6.** Docking pose of metronidazole in β-tubulin. A) 3D and B) 2D representation with residue contacts.	**4**
**Table S2.** Docking scores of the top five poses of metronidazole and the most active compounds in the proposed binding site of PFOR.	**5**
**Table S3.** Docking scores of the top five poses of metronidazole and the most active compounds in the proposed binding site of β-tubulin.	**6**

```
CLUSTAL O(1.2.4) multiple sequence alignment


sp|P05304|TBB_GIAIN          MREIVHIQAGQCGNQIGAKFWEVISDEHGVDPSGEYRGDSELQIERINVYFNEAAGGRYV       60
tr|W5Q039|W5Q039_SHEEP       MREIVHIQAGQCGNQIGAKFWEVISDEHGIDPSGNYVGDSDLQLERISVYYNEASSHKYV       60
sp|Q6B856|TBB2B_BOVIN        MREIVHIQAGQCGNQIGAKFWEVISDEHGIDPTGSYHGDSDLQLERINVYYNEATGNKYV       60
sp|P02554|TBB_PIG            MREIVHIQAGQCGNQIGAKFWEVISDEHGIDPTGSYHGDSDLQLERINVYYNEAAGNKYV       60
                             *****************************:**:*.* ***:**:***.**:***:. :**

sp|P05304|TBB_GIAIN          PRAILVDLEPGTMDSVRAGPFGQIFRPDNFVFGQSGAGNNWAKGHYTEGAELVDAVLDVV       120
tr|W5Q039|W5Q039_SHEEP       PRAILVDLEPGTMDSVRSGAFGHLFRPDNFIFGQSGAGNNWAKGHYTEGAELVDSVLDVV       120
sp|Q6B856|TBB2B_BOVIN        PRAILVDLEPGTMDSVRSGPFGQIFRPDNFVFGQSGAGNNWAKGHYTEGAELVDSVLDVV       120
sp|P02554|TBB_PIG            PRAILVDLEPGTMDSVRSGPFGQIFRPDNFVFGQSGAGNNWAKGHYTEGAELVDSVLDVV       120
                             *****************:* **::******:***********************:*****

sp|P05304|TBB_GIAIN          RKRSEACDCLQGFQICHSLGGGTGAGMGTLLIAKIREEYPDRMMCTFSVVPSPKVSDTVV       180
tr|W5Q039|W5Q039_SHEEP       RKECENCDCLQGFQLTHSLGGGTGSGMGTLLISKVREEYPDRIMNTFSVVPSPKVSDTVV       180
sp|Q6B856|TBB2B_BOVIN        RKESESCDCLQGFQLTHSLGGGTGSGMGTLLISKIREEYPDRIMNTFSVMPSPKVSDTVV       180
sp|P02554|TBB_PIG            RKESESCDCLQGFQLTHSLGGGTGSGMGTLLISKIREEYPDRIMNTFSVVPSPKVSDTVV       180
                             **..* ********: ********:*******:*:*******:* ****:**********

sp|P05304|TBB_GIAIN          EPYNATLSVHQLVEHADEVFCIDNEALYDICFRTLKLTCPTYGDLNHLVSLVMSGCTSCL       240
tr|W5Q039|W5Q039_SHEEP       EPYNATLSIHQLVENTDETYCIDNEALYDICFRTLKLATPTYGDLNHLVSATMSGVTTSL       240
sp|Q6B856|TBB2B_BOVIN        EPYNATLSVHQLVENTDETYCIDNEALYDICFRTLKLTTPTYGDLNHLVSATMSGVTTCL       240
sp|P02554|TBB_PIG            EPYNATLSVHQLVENTDETYCIDNEALYDICFRTLKLTTPTYGDLNHLVSATMSGVTTCL       240
                             ********:*****::**.:*****************: *********** .*** *:.*

sp|P05304|TBB_GIAIN          RFPGQLNADLRKLAVNLIPFPRLHFFLVGFAPLTSRGSQIYRALTVPELVSQMFDNKNMM       300
tr|W5Q039|W5Q039_SHEEP       RFPGQLNADLRKLAVNMVPFPRLHFFMPGFAPLTAVPGAEYRALTVPELTQQMFDAKNMM       300
sp|Q6B856|TBB2B_BOVIN        RFPGQLNADLRKLAVNMVPFPRLHFFMPGFAPLTSRGSQQYRALTVPELTQQMFDSKNMM       300
sp|P02554|TBB_PIG            RFPGQLNADLRKLAVNMVPFPRLHFFMPGFAPLTSRGSQQYRALTVPELTQQMFDAKNMM       300
                             ****************::********: ******:  .  *********..**** ****

sp|P05304|TBB_GIAIN          AASDPRHGRYLTAAAMFRGRMSTKEVDEQMLNIQNKNSSYFVEWIPNNMKVSVCDIPPRG       360
tr|W5Q039|W5Q039_SHEEP       AACDPRHGRYLTVATVFRGRMSMKEVDEQMLAIQSKNSSYFVEWIPNNVKVAVCDIPPRG       360
sp|Q6B856|TBB2B_BOVIN        AACDPRHGRYLTVAAIFRGRMSMKEVDEQMLNVQNKNSSYFVEWIPNNVKTAVCDIPPRG       360
sp|P02554|TBB_PIG            AACDPRHGRYLTVAAVFRGRMSMKEVDEQMLNVQNKNSSYFVEWIPNNVKTAVCDIPPRG       360
                             **.*********.*::****** ******** :*.*************:*.:********

sp|P05304|TBB_GIAIN          LKMAATFIGNSTCIQELFKRVGEQFSAMFRRKAFLHWYTGEGMDEMEFTEAESNMNDLVS       420
tr|W5Q039|W5Q039_SHEEP       LKMSSTFIGNSTAIQELFKRISEQFTAMFRRKAFLHWYTGEGMDEMEFTEAESNMNDLVS       420
sp|Q6B856|TBB2B_BOVIN        LKMSATFIGNSTAIQELFKRISEQFTAMFRRKAFLHWYTGEGMDEMEFTEAESNMNDLVS       420
sp|P02554|TBB_PIG            LKMSATFIGNSTAIQELFKRISEQFTAMFRRKAFLHWYTGEGMDEMEFTEAESNMNDLVS       420
                             ***::*******.*******:.***:**********************************

sp|P05304|TBB_GIAIN          EYQQYQEAGVDEGEEFEEEEDFGDEQ  446
tr|W5Q039|W5Q039_SHEEP       EYQQYQDATAEEEGEMYEDDEEESEA  446
sp|Q6B856|TBB2B_BOVIN        EYQQYQDATADEQGEFEEEEGEDEA-  445
sp|P02554|TBB_PIG            EYQQYQDATADEQGEFEEEGEEDEA-  445
                             ******:* .:*  *: *:     .
```

Figure S1. Multiple sequence alignment of β-tubulin from different organisms used in this study.

Table S1. Template scoring as obtained by PSI-BLAST and YASARA.

Template	Total Score	BLAST E-value	Align Score	Cover	ID	Resolution	Covered residues	Quality Score
1	1685.11	0	2049	96%	5M7E-D	2.05	427	0.859
2	1649.22	0	2053	95%	5JQG-B	2.24	423	0.847
3	1609.41	0	2031	97%	4LNU-B	2.19	431	0.82
4	1601.17	0	2038	97%	3RYC-D	2.10	431	0.813
5	1598.02	0	2043	96%	6H9B-D	2.75	427	0.817

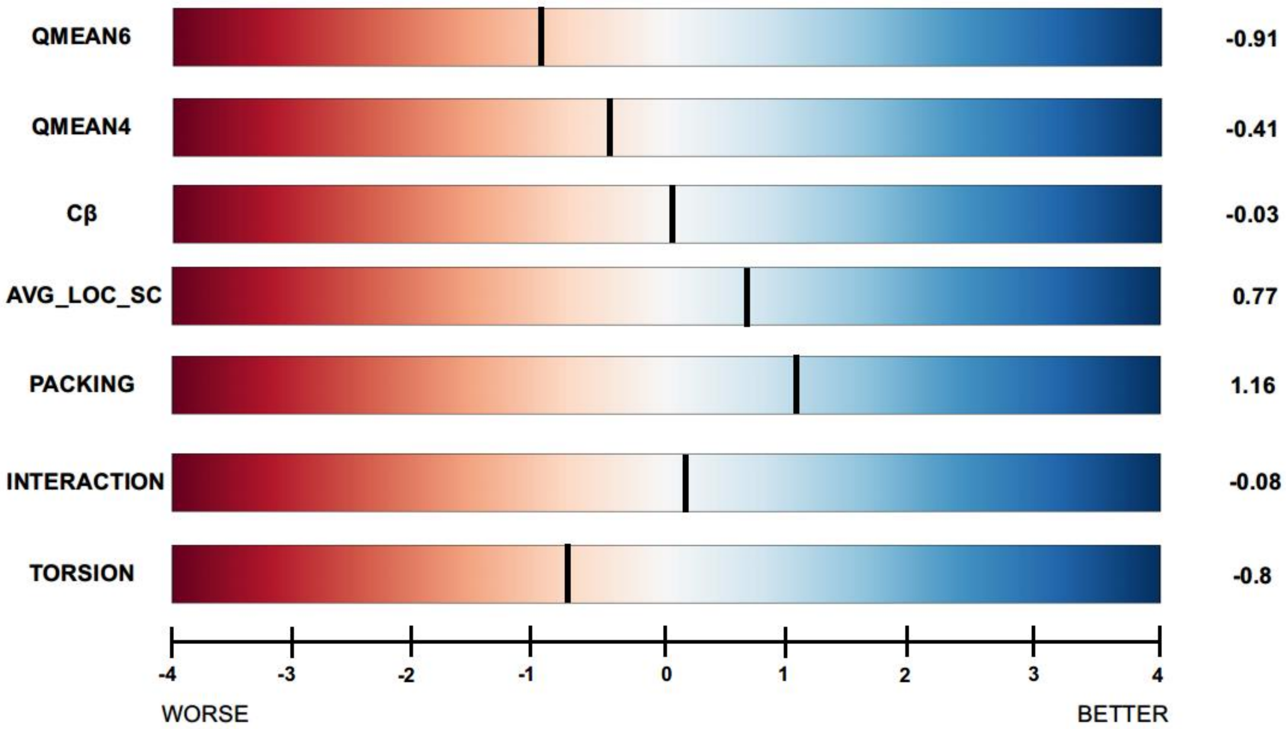

Figure S2. Global quality measures of the homology model constructed for *G. duodenalis* β-tubulin.

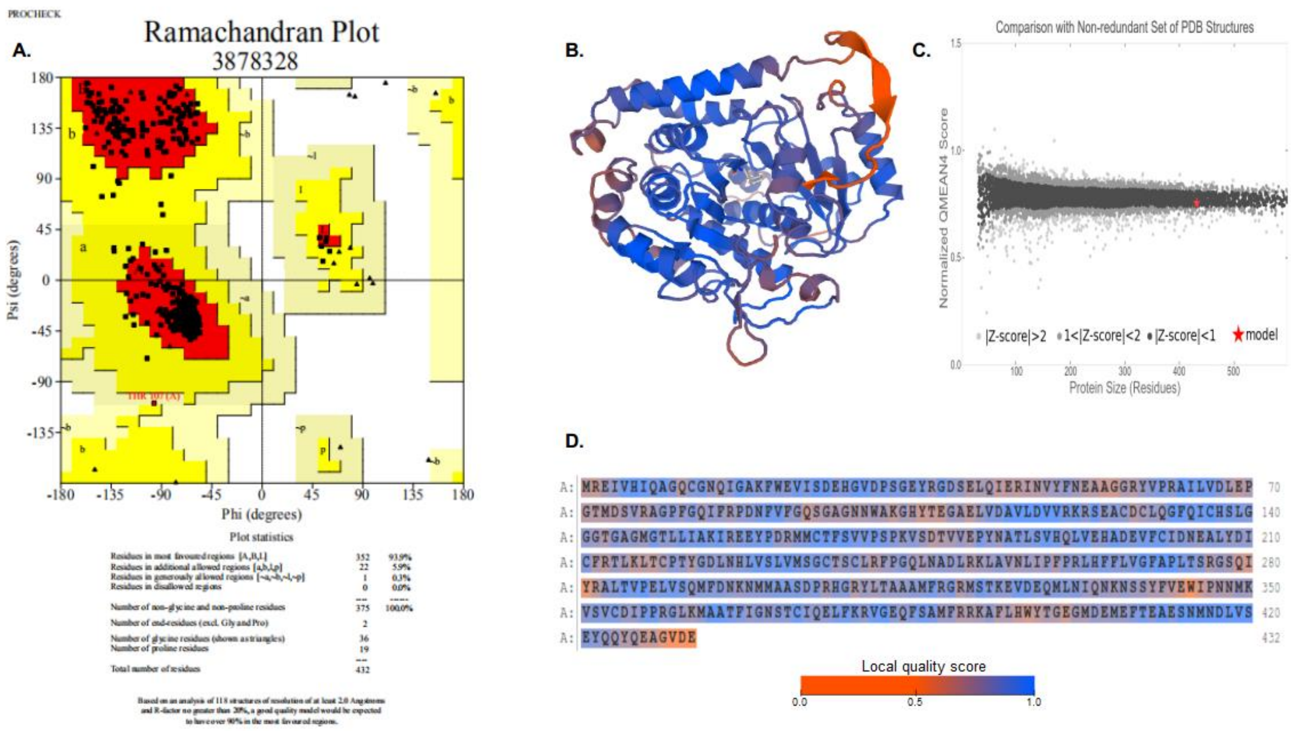

Figure S3. Additional quality assessment of the homology model. (A) Main Ramachandran plot. (B) 3D structure of homology model colored by quality. (C) QMEAN4 normalized score. (D) Local quality per-residue.

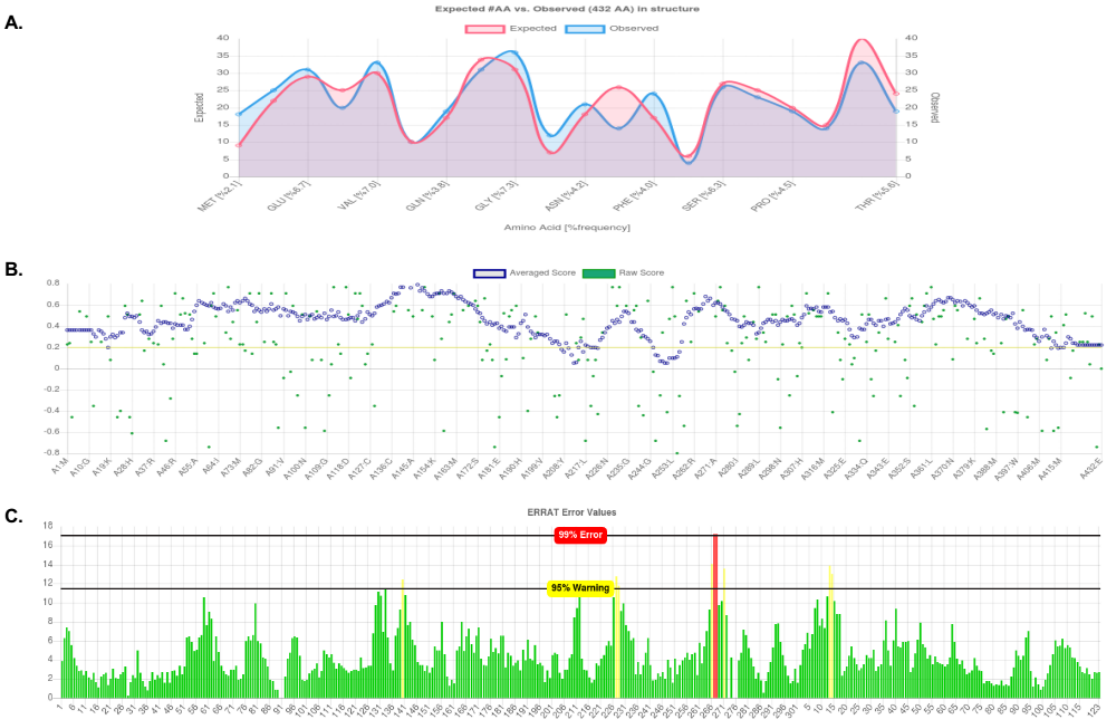

Figure S4. Model quality obtained from SAVES server. (A) Residue distribution. (B) Verify 3D. (C) ERRAT.

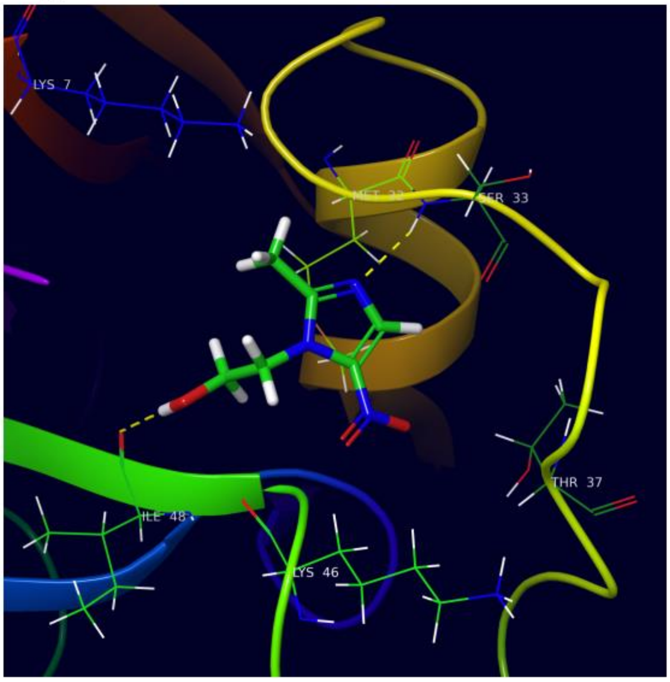

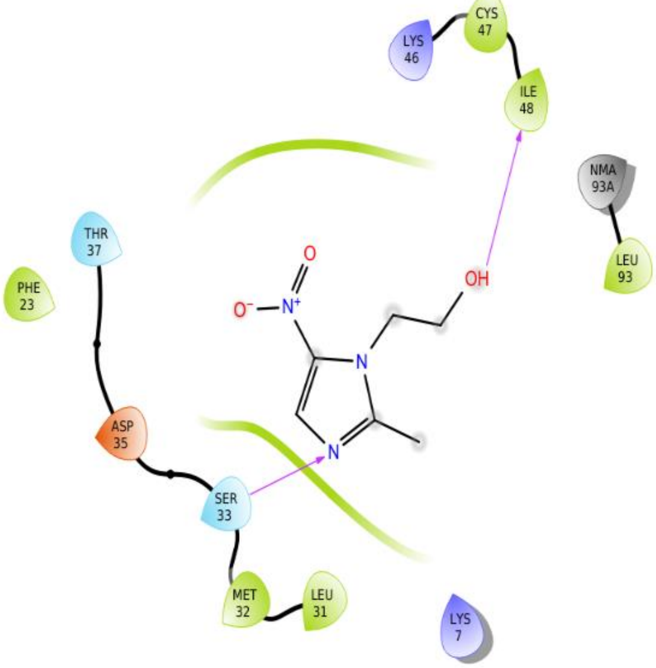

Figure S5. Docking pose of metronidazole in PFOR. (A) 3D and (B) 2D representation with residue contacts.

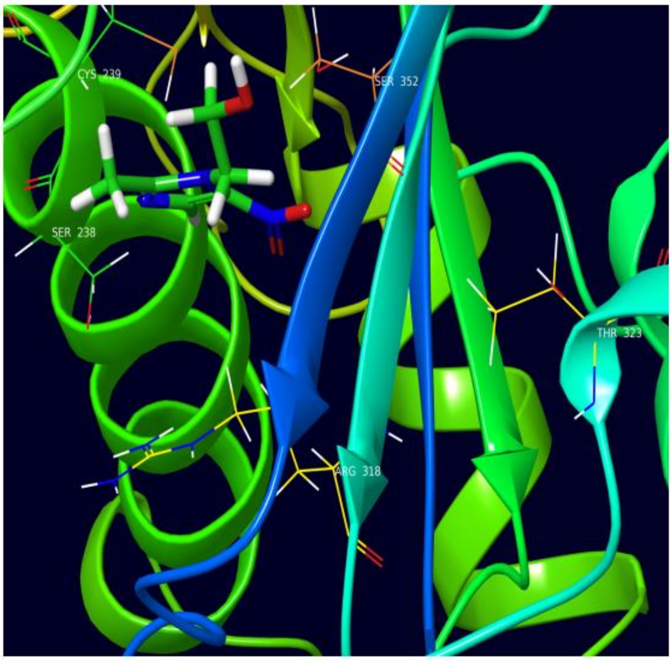

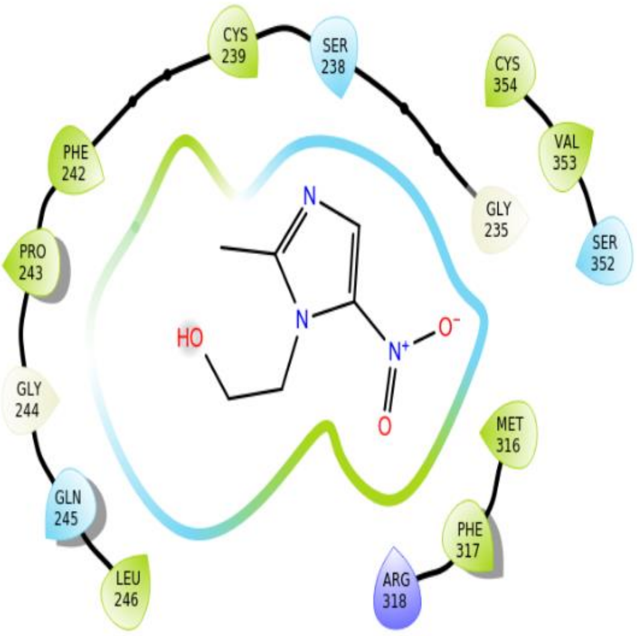

Figure S6. Docking pose of metronidazole in β-tubulin. (A) 3D and (B) 2D representation with residue contacts.

Table S2. Docking scores of the top five poses of metronidazole and the most active compounds in the proposed binding site for PFOR.

Compound	Scoring
Metronidazole	-5.00
	-4.96
	-4.85
	-4.83
	-4.80
1	-6.20
	-6.10
	-6.07
	-6.01
	-5.99
2	-5.70
	-5.48
	-5.47
	-5.47
	-5.46

Table S3. Docking scores of the top five poses of nocodazole and the most active compounds in the proposed binding site of β-tubulin.

Compound	Scoring
Metronidazole	-4.82
	-4.76
	-4.72
	-4.70
	-4.68
1	-6.25
	-5.83
	-5.41
	-5.16
	-5.12
2	-6.33
	-6.31
	-6.01
	-5.96
	-5.80
